# Assessment of antioxidant enzyme activities in erythrocytes of pre-hypertensive and hypertensive women

**Published:** 2010

**Authors:** Farshad Amirkhizi, Fereydoun Siassi, Mahmoud Djalali, Abbas Rahimi Foroushani

**Affiliations:** a Department of Nutrition, Faculty of Health, Zabol University of Medical Sciences, Zabol, Iran; b Department of Nutrition and Biochemistry, Faculty of Public Health, Tehran University of Medical Sciences, Tehran, Iran; c Department of Epidemiology and Biostatistics, Faculty of Public Health, Tehran University of Medical Sciences, Tehran, Iran

**Keywords:** High Blood Pressure, Antioxidants, Biocatalysts, Woman

## Abstract

**BACKGROUND:**

Few studies that have investigated hypertension have considered a state of oxidative stress that can contribute to the development of atherosclerosis and other hypertension induced organ damage. The aim of this study was to investigate whether pre-hypertension and hypertension status is associated with activities of erythrocyte antioxidant enzymes in a random sample of cardiovascular disease-free women.

**METHODS:**

In this case-control study, 53 pre-hypertensive women, 32 hypertensive women and 75 healthy controls were included. General information was gathered using questionnaires and face-to-face interviews. Blood pressure and anthropometric measurements were measured for each subject. Venous blood samples were drawn from subjects and plasma was separated. Activities of erythrocyte antioxidant enzymes were also evaluated by measuring activities of copper zinc-superoxide dismutase (CuZn-SOD), glutathione peroxidase (GPX) and catalase (CAT) in selected subjects.

**RESULTS:**

Fifty-three (33.1%) and 32 (20%) participants were pre-hypertensive and hypertensive, respectively. The hypertensive and pre-hypertensive women had lower CuZn-SOD (p < 0.001) and GPX (p < 0.01) activities compared to normotensives. Furthermore, hypertensive women had lower CAT activity compared to pre-hypertensive and normotensive women (p < 0.001). Moreover, significant differences were also observed between hypertensive and pre-hypertensive women in erythrocyte CAT activity (p < 0.01).

**CONCLUSIONS:**

The present findings show that activities of erythrocyte antioxidant enzymes decrease in pre-hypertensive and hypertensive women, which may eventually lead to atherosclerosis and other high blood pressure related health problems.

Hypertension is a common health problem in developed countries.^1^ Untreated high blood pressure leads to many degenerative diseases, including heart failure, renal disease and coronary heart disease.^2^

Several theories have been tested to explain how elevated blood pressure might confer the increased risk of cardiovascular disease (CVD). For example, inflammation of arterial wall, endothelial dysfunction and increased lipid peroxidation (as marker of oxidative stress) have been implicated in hypertension.^2^–^4^

Based on recent report of the Seventh Report of the Joint National Committee on Prevention, Detection, Evaluation and Treatment of High Blood Pressure (JNC VII), individuals who had systolic blood pressure between 120 and 139 mmHg or diastolic blood pressure between 80 and 89 mmHg are categorized as pre-hypertensive.^5^ In other words, this is the group at high risk for developing essential hypertension and CVD.

Some clinical studies have shown increased reactive oxygen species (ROS) production in hypertension.^6^ These ROS are formed via leakage from the mitochondrial electron transport chain in all cells during normal aerobic metabolism.^7^ Several antioxidant enzymes such as copper zinc superoxide dismutase (CuZn-SOD), glutathione peroxidase (GPX) and catalase (CAT) have been reported which have the ability of detoxification of ROS including superoxide anion (O_2_^-^) and hydrogen peroxide (H_2_O_2_).^8^

There is growing evidence that oxidative stress contributes to the pathogenesis of hypertension, but the mechanisms contributing to increased free radical production in hypertension are not well understood. In a previous study by the present group, it was observed that oxidative stress by-products increase in pre-hypertension and hypertension status.^9^

Some studies have shown that an increase in the activity of pro-oxidant enzymes, mainly NADPH oxidase, has been implicated in the high level of ROS in several cellular models of hypertensive subjects.^10^,^11^

On the other hand, decreased activities of antioxidant enzymes, including CuZn-SOD, GPX and CAT may also contribute to oxidative stress. However, the importance of abnormalities in antioxidant mechanisms has been largely neglected, and whether or not the anti-oxidant enzymes activities decrease in pre-hypertension has not been established. Therefore, the objective of this study was to determine whether or not the high oxidative stress levels observed in hypertensive subjects is in part dependent on defective erythrocyte anti-oxidant enzyme activities.

In the present study, the activities of main antioxidant enzymes, CuZn-SOD, GPX and CAT in erythrocytes of pre-hypertensive and hypertensive women were analyzed. Perhaps, findings of this study could cast some light on the mechanism underlying the increased risk of cardiovascular diseases in subjects with pre-hypertensive and hypertensive blood pressure levels.

## Methods

The subjects selected for this study were recruited from women receiving the services of rural health centers of Kerman Province, Iran. In this study, 160 women of 20-45 years old were randomly selected. The including criteria were: 1) not being pregnant and lactating, 2) not having any kind of cancer, cardiovascular, diabetes, renal and liver diseases, and 3) not taking any vitamin or mineral supplements.

All participants were informed about the aims and procedure of the study and signed written consent. General data were gathered from samples using questionnaires and face-to-face interviews. Data collecting form included demographic characteristics (age, number of pregnancies and education), and detailed medical history and lifestyle habits, such as smoking status and physical activity. Physical activity was classified as active if subjects reported “moving, walking and working energetically and participating in vigorous exercise”; otherwise, they were classified as inactive.

Body weight was measured while the subjects were wearing light clothing without shoes to the nearest 0.1 Kg. Height was measured to the nearest 0.5 cm, without shoes in standing position. Body mass index (BMI) was calculated as weight (in kilogram) divided by height (in meters-squared). In the current study, general obesity was defined as BMI ≥ 30 kg/m^2^·^12^ Waist circumferences (cm) were measured to the nearest 0.1 cm using an unstretched tape measure. The waist-to-hip ratio was then calculated. Waist circumference (WC) greater than 88 cm was considered an indicator of abdominal obesity.^13^

Arterial blood pressure (BP) was measured with a mercury sphygmomanometer. All subjects were at least 15 minute at rest in a quiet environment. Blood pressure measurements were taken at the right arm relaxed and well supported by a table, with an angle 45° from the trunk. Systolic blood pressure (SBP) and diastolic blood pressure (DBP) were averaged by using three readings measured at 5 minute intervals. Differences of < 5 mmHg were allowed. Study participants were divided into three groups according to their average SBP and DBP levels. Subjects whose average BP levels were greater or equal to 140 mmHg/90 mmHg or were under antihypertensive medication or physician has told them that they have hypertension but they were untreated were classified as hypertensive. Participants who had mean systolic/diastolic blood pressures within the range of 120-139 mmHg/80-89 mmHg and they had never been told that they have high BP levels are defined as pre-hypertensive as it has recently suggested by the Seventh Report of the Joint National Committee on the Prevention Detection, Evaluation, and Treatment of High Blood Pressure (JNC VII).^5^ Fifty-three women were recruited as a pre-hypertensive group and 75 healthy, normotensive women were selected as a control group. Thirty-two women were also recruited as hypertensive group.

A validated food-frequency questionnaire (FFQ) was used for assessing usual dietary intakes.^14^ A trained dietitian administered all questionnaires. Participants reported their frequency of consumption of a given serving of each food item during the previous year on a daily, weekly, or monthly basis. For analysis, daily intake of all food items from FFQ was computed and then consumed foods were converted to grams using household measures. The Food Processor^®^Program (Version 2) was used to analyze the data from the FFQ.

Non-fasting blood samples were obtained from median cubital vein between 8.00 and 12.00 A.M. and collected into standard tubes containing ethylene diamine tetra acetic acid (EDTA). Blood samples centrifuged at 3000 rpm for 15 minutes at 4°C and plasma was separated. The buffy coat was removed and remaining erythrocytes were washed three times in cold salin (9.0 g/l NaCl) and hemolysed by the addition of an equal volume of ice-cold demineralized ultrapure water. Subjects’ hemolysates were stored in -70°C until analysis.

In order to express the enzymes’ activities per gram hemoglobin (Hb), Hb concentration was measured in the hemolysates with a standard kit (Zist chemistry Laboratories, Iran) using the cyanmethemoglobin method (Drabkin’s method).

Catalase activity was determined by method of Hugo Aebi.^15^ Activity of CAT was determined by following the decomposition of H_2_O_2_ in phosphate buffer pH 7.2 spectrophotometrically at 230 nm.

Glutathione peroxidase (GPX) activity was measured according to Paglia and Valentine method.^16^ Measurement of copper zinc-superoxide dismutase (CuZn-SOD) activity was performed by Ransod kit (Randox Laboratories, cat. no.SD-125).

Continuous variables are presented as mean ± standard deviation, while categorical variables are presented as absolute and relative frequencies. Kolmogorov-Smirnov test was performed to test their normal distribution. Differences between study groups were compared by one-way analysis of variance (ANOVA) for continuous data and the Chi square for categorical data. Post hoc comparisons were performed with Tukey test.

Correlations between normally distributed continuous variables were evaluated by the calculation of Pearson’s correlation coefficients. Furthermore, multivariate adjusted means of enzyme activities were computed using analysis of covariance (ANCOVA) in 3 different models. The first model adjusted for age (y). It was further adjusted for physical activity (yes or no), number of pregnancies and energy intake (kcal) in the 2^nd^ model. In the 3^rd^ model, BMI and WC were controlled.

Multiple linear regression analysis was applied to evaluate the association between anti-oxidant enzyme activities with hypertension status of the participants, after controlling for various potential confounders. Also the adjusted R^2^ was calculated in order to find how well each fitted model predicted the dependent variables. A value of p < 0.05 was considered to be statistically significant.

## Results

Fifty three (33.1%) and thirty two (20%) participants were pre-hypertensive and hypertensive, respectively. characteristics of the study participants by blood pressure status are shown in table 1. Age- and energy-adjusted means for dietary variables are also presented in this table. Compared with pre-hypertensive and hypertensive women, individuals in normotensive group were younger (p = 0.001). Additionally, the prevalence of general and abdominal obesity were higher in hypertensive and pre-hypertensive participants (p = 0.002).

**Table 1:** Characteristics and dietary intakes of the participants by blood pressure status

Variables	Normotensive (n = 75)	Pre-hypertensive (n = 53)	Hypertensive (n = 32)	P value†
Age (years)	31 ± 11	36 ± 12	42 ± 3*	0.001
Education status (years of school)	7 ± 4	7 ± 3	6 ± 3	0.321
Physical inactivity (%)	48	47	51	0.247
Body mass index (Kg/m2)	24 ± 4	26 ± 5*	29 ± 4**	0.001
Obesity (%)	9	12*	16**	0.002
Waist-to hip ratio	0.74 ± 0.06	0.82 ± 0.08	0.91 ± 0.07*	0.03
Abdominal obesity (%)	11	14*	19*	0.002
Number of pregnancies	3.4 ± 2.1	3.8 ± 3.1	4.2 ± 3.7*	0.031
**Dietary intakes:**				
Total energy (kcal/day)	2078 ± 241	2116 ± 341	2287 ± 357	0.453
Carbohydrate (% total energy)	54.6 ± 4.1	53.7 ± 3.5	54.6 ± 5.4	0.367
Protein (% total energy)	14 ± 3.7	13 ± 4.1	14 ± 3.6	0.264
Fat (% total energy)	31 ± 3.4	32 ± 3.9	34 ± 34.3	0.358

† P value derived from one-way ANOVA that used to evaluate differences in the investigated variables and blood pressure

* P < 0.05 from the post hoc comparisons (Tukey test) of the investigated variables between pre-hypertensive or hypertensive subjects compared to normotensive

** P < 0.01 from the post hoc comparisons (Tukey test) of the investigated variables between pre-hypertensive or hypertensive subjects compared to normotensive

Erythrocyte CuZn-SOD activity was inversely correlated with age (r = -0.218, p < 0.01), BMI (r = -0.325, p < 0.01), SBP (r = -0.279, p < 0.01) and DBP(r = -0.295, p < 0.01). Furthermore, erythrocyte GPX activity was inversely correlated with BMI (r = -0.426, p < 0.001), WC (r = -0.312, p < 0.001) and DBP (r = -0.376, p < 0.001). Regarding erythrocyte CAT activity, it was found that it is inversely correlated with SBP (r = -0.243, p < 0.01) and DBP (r = -0.318, p < 0.001). Because erythrocyte anti-oxidant enzyme activities correlated with age, BMI and WC, thus, all further analyses were adjusted for these confounders. Multivariate adjusted means for antioxidant enzyme activities by blood pressure status are presented in table 2. After controlling age, the hypertensive and pre-hypertensive women had lower CuZn-SOD and GPX activities compared to normotensives. Furthermore, hypertensive women had lower CAT activity compared to pre-hypertensive and normotensive women. Moreover, significant differences were also observed between hypertensive and pre-hypertensive women in erythrocyte CAT activity. These associations remained significant even after additional control for other confounders. However, adjustment for BMI and WC attenuated all associations.

**Table 2:** Multivariate adjusted means for antioxidant enzyme activities of the participants by blood pressure status†

Enzyme activities	Normotensive (n = 75)	Pre-hypertensive (n = 53)	Hypertensive (n = 32)	P value††
**CuZn-SOD (U/gHb):**				
Model I‡	962 ± 86	621 ± 71*	547 ± 62*	< 0.001
Model II‡‡	976 ± 71	618 ± 59*	552 ± 58*	< 0.001
Model III‡‡‡	982 ± 76	674 ± 61**	586 ± 51**	< 0.01
**GPX (U/gHb):**				
Model I	128.2 ± 56.4	74.7 ± 41.8**	72.8 ± 46.6**	< 0.01
Model II	131.5 ± 49.3	112.4 ± 36.7	69.3 ± 39.8**	< 0.01
Model III	136. ± 52.1	116.2 ± 41.3	76.3 ± 36.4**	< 0.05
**CAT (K/gHb):**				
Model I	367.6 ± 52.6	326.6 ± 48.6***	164.8 ± 40.2*	< 0.001
Model II	374.3 ± 49.7	331.2 ± 51.7***	198.1 ± 46.8*	< 0.001
Model III	378.2 ± 56.1	269.4 ± 45.6	236.6 ± 42.7**	< 0.01

† Data are mean ± SD

†† P values are based on analysis of covariance (ANCOVA)

‡ Model I, adjusted for age

‡‡ Model II, adjusted for physical activity (yes or no), number of pregnancies and energy intake (kcal)

‡‡‡ Model III, additionally adjusted for BMI and WC

* P < 0.01 from the post hoc comparisons (Tukey test) between pre-hypertensive or hypertensive vs normotensive participants

** P < 0.001 from the post hoc comparisons (Tukey test) between pre-hypertensive or hypertensive vs normotensive participants

*** P < 0.01 from the post hoc comparisons (Tukey test) between pre-hypertensive and hypertensive participants

The results of multiple linear regression models, which included antioxidant enzyme activities as dependent and hypertension status as independent variables, are shown in table 3. It was observed that the aforementioned relationships remained significant even after adjusting for the previous characteristics of the participants (Table 3). Therefore, erythrocyte antioxidant enzyme activities were independently associated with hypertension status.

**Table 3:** Results from multiple linear regression analysis that evaluated the association between antioxidant enzyme activities (dependent) and hypertension status (independent), after controlling for various potential confounders

Model for	Beta-coefficient ± SE	P value
**CuZn-SOD activity:**		
Constant of the model	52 ± 3	
Pre-hypertension vs. normal	-8.3 ± 2.6	0.04
Hypertension vs. normal	-12.7 ± 3.8	0.03
Age (year)	-0.62 ± 0.12	0.01
Physical activity (yes vs. no)	0.29 ± 0.06	N.S*
Number of pregnancies	-2.1 ± 0.8	N.S
Body mass index (Kg/m2)	-3.6 ± 1.2	0.005
Waist-to hip ratio	-2.6 ± 0.4	0.002
Adjusted R2 with/without hypertension status	9.4%/7.3%	
**GPX activity:**		
Constant of the model	76 ± 13	
Pre-hypertension vs. normal	-12.8 ± 4.2	0.02
Hypertension vs. normal	-14.6 ± 5.2	0.004
Age (year)	-0.32 ± 0.06	N.S
Physical activity (yes vs. no)	3.8 ± 0.2	N.S
Number of pregnancies	-0.72 ± 0.06	N.S
Body mass index (Kg/m2)	-1.54 ± 0.2	0.003
Waist-to hip ratio	-2.32 ± 0.4	0.001
Adjusted R2 with/without hypertension status	8.7%/10.4%	
**CAT activity:**		
Constant of the model	64 ± 8	
Pre-hypertension vs. normal	-14.6 ± 6.4	0.005
Hypertension vs. normal	-17.3 ± 6.6	0.001
Age (year)	0.54 ± 0.07	N.S
Physical activity (yes vs. no)	4.6 ± 0.4	N.S
Number of pregnancies	-0.52 ± 0.08	N.S
Body mass index (Kg/m2)	2.68 ± 1.3	N.S
Waist-to hip ratio	3.46 ± 0.54	N.S
Adjusted R2 with/without hypertension status	10.2%/8.7%	

* Non-significant

## Discussion

This study reveals the decreased activity of main antioxidant enzymes in pre-hypertensive and hypertensive women, without any clinical evidence of cardiovascular disease. A consistent relationship between hypertension status and antioxidant enzyme activities, after adjusting various potential confounders was found particularly. The findings support the hypothesis that pre-hypertensive and hypertensive subjects are under decreased activity of antioxidant enzymes.

Oxidative stress seems to play an important role in the pathophysiology of essential hypertension,^17^ other than hypertension induced organ damage.^18^ In one of the previous studies by the present group,^9^ it was also shown that pre-hypertensive women had higher oxidative stress and lower total antioxidant capacity (TAC) compared to normotensives. Increased production of free radicals and the declining activity of the antioxidant defense system are two possible factors which may lead to increased oxidative stress.

Antioxidant enzymes are the major defense system of cells in normal aerobic reactions,^19^ and erythrocytes are equipped with a highly effective antioxidant defense system.^20^ Although, previous studies have investigated the antioxidant enzyme activities in hypertension,^21^,^22^ but to our knowledge, no previous study has investigated the activities of these enzymes in pre-hypertension. In present study, the lower enzymatic activity of CuZn-SOD in hypertensive and pre-hypertensive women compared to normatensives was found. Reported SOD activity in plasma,^23^ as well as in erythrocytes^20^ and mononuclear cells^24^ was significantly lower in hypertensive subjects.

Numerous studies have been done on relationship between hypertension and activity of antioxidant enzymes but due to the conflicting results, the question of whether hypertension or oxidative stress is the primary event is still a matter of debate.

Moreover, it is uncertain whether the reduction of antioxidant enzyme activities in pre-hypertension is due to decreased enzymes activity by increasing oxidative stress or not. However, it has been reported that damage of proteins increased in patients with essential hypertension.^23^ The consequences of such oxidative protein damage in high blood pressure status may be impaired enzymatic activity. However, since oxidative protein damage was not assessed in the present subjects; it is unknown whether this process was increased in the borderline blood pressure level (pre-hypertension). However impaired CuZn-SOD activity, the first line of defense against ROS in vascular wall,^25^ has been reported in increased oxidative stress.^26^ In addition to the decrease of CuZn-SOD activity (as first line of defense against ROS), production of superoxide anion elevated in hypertension due to increased activation of NADH or NADPH oxidase.^27^ Thus, suppose that in such conditions excess of superoxide anion escape antioxidant defense and contribute to decreased nitrite oxide (NO) bioavailability and consequent endothelial dysfunction, already documented in high blood pressure.^28^ Although, by evaluating the erythrocyte CuZn-SOD activity in this study, only a limited part of total vascular SOD was estimated, and it is believed that the lower activities observed in pre-hypertensive and hypertensive women are still representative of the situation in vascular wall.

Interestingly, the pattern of changes observed in erythrocyte GPX activity in study groups resembles that of CuZn-SOD (Table 2). In accordance with the present findings, lower GPX activity has been reported in hypertension by previous studies.^21^,^22^ Inversely, Simic et al reported increased plasma GPX activity in patients with essential hypertension.^23^ These controversial findings observed between the present results and Simic et al study are not unexpected, as one is active in plasma and other in erythrocytes. However, these controversial findings may be partially due to differences in stage of hypertension, laboratory methods used and/or sample size. Although the data on regulation erythrocyte GPX activities are still lacking, it is supposed that decreased erythrocyte GPX activities may be due more sensitively to being damaged by oxidative stress. If this is coupled with an increase in ROS-generating process, the consequence is a deep redox disturbance.

Interestingly, erythrocyte CAT activities are decreased in a manner which parallels the severity of hypertension as measured by blood pressure. The negative relationship which was observed in the present study between CAT activity and blood pressure indicators (SBP and DBP) was confirmed this finding. Moreover, elevated plasma hydrogen peroxide, a major CAT substrate, has also been reported in hypertensive subjects.^27^ If this ROS is not scavenged, it may lead to lipid peroxidation in arterial wall, which correlated with increased blood pressure.^27^ Furthermore, a marked decreased in plasma CAT activity has been reported in hypertensive subjects.^21^,^23^ Since, the majority of plasma CAT is released from erythrocytes,^29^ therefore decreased plasma CAT might be partially linked to a decrease in erythrocyte CAT. Although, intracellular CAT is very important for scavenging of hydrogen peroxide, the precise mechanisms underlying the affect and its activity is not yet fully clarified.

In the present study, the negative correlation between erythrocyte antioxidant enzyme activities with pre-hypertension status, as well as with age and BMI was observed. The observations of Rao et al^30^ and Ji et al^31^ confirm the present findings. These investigators have reported the prominent risk factors associated with cardiovascular disease (like obesity and increased age) inversely correlated with anti-oxidant enzyme activities in the kidney, brain and heart. Therefore, some cardiovascular related factors such as obesity and aging might have a significant impact between oxidation process and pre-hypertension status. Surprisingly, it was also found that even slightly elevated blood pressure, in the pre-hypertension stage are associated with significant lower antioxidant enzyme activities, independently of the coexistence of other atherogenic risk factors.

The present study may have some limitations in data gathering like all cross-sectional studies. First, as with all observational studies, these results could be biased by unrecognized confounders. Second, the cross-sectional study does not allow us to conclude causal relationships. Third, couldn’t assess nutrient intakes (including vitamins and minerals) of participants. Other limitations of this study were the limited number of subjects, and the fact that only women were included in the analysis.

## Conclusions

In conclusion, activities of erythrocyte antioxidant enzyme in pre-hypertensive and hypertensive women were studied and their activities were compared with normotensive subjects, independently of other coexisting risk factors or unhealthy lifestyle behaviors.

The present study provides further evidence suggesting that the high blood pressure leads to decreased antioxidant enzyme activities, which in turn, may contribute to oxidative stress related diseases like atherosclerosis and cardiovascular disease. Further studies are necessary to delineate the factors involved in the disturbed regulation of the erythrocyte antioxidant enzyme activities in these levels of blood pressure.

## References

[1] Medina-Lezama J, Zea-Diaz H, Morey-Vargas OL, Bolanos-Salazar JF, Postigo-MacDowall M, Paredes-Diaz S (2007). Prevalence and patterns of hypertension in Peruvian Andean Hispanics: the PREVENCION study. J Am Soc Hypertens.

[2] Hartford M, Wikstrand JC, Wallentin I, Ljugman SM, Berglund GL (1985). Left ventricular wall stress and systolic function in untreated primary hypertension. Hypertension.

[3] Palmer A, Bulpitt C, Beevers G, Coles E, Fletcher A, Lendingham J (1995). Risk factors for ischaemic heart disease and stroke mortality in young and old hypertensive patients. J Hum Hypertens.

[4] Maggi E, Marchesi E, Ravetta V, Falaschi F, Finardi G, Bellomo G (1993). Low density lipoprotein oxidation in essential hypertension. J Hypertens.

[5] Lenfant C, Chobanian AV, Jones DW, Roccella EJ (2003). Seventh report of the Joint National Committee on the Prevention, Detection, Evaluation and Treatment of High Blood Pressure (JNC 7): resetting the hypertension sails. Hypertension.

[6] Touyz RM (2004). Reactive oxygen species, vascular oxidative stress, and redox signaling in hypertension: what is the clinical significance?. Hypertension.

[7] McCord JM (1993). Human disease, free radicals, and the oxidant/antioxidant balance. Clin Biochem.

[8] Michiels C, Raes M, Toussaint O, Remacle J (1994). Importance of Seglutathione peroxidase, catalase, and Cu/Zn-SOD for cell survival against oxidative stress. Free Radic Biol Med.

[9] Amirkhizi F, Siassi F, Djalali M, Minaie S, Dorosty AR (2007). Assessment of oxidative stress markers related to atherosclerosis in pre-hypertensive women. J Tehran Univ Heart Ctr.

[10] Nakazono K, Watanabe N, Matsuno K, Sasaki J, Sato T, Inoue M (1991). Does superoxide underlie the pathogenesis of hypertension?. Proc Natl Acad Sci U S A.

[11] Touyz RM, Tabet F, Schiffrin EL (2003). Redox-dependent signaling by angiotensin II and vascular remodeling in hypertension. Clin Experiment Pharmacol Physiol.

[12] (2000). Obesity: preventing and managing the global epidemic. Report of a WHO consultation.

[13] (2001). Expert Panel on Detection, Evaluation and Treatment of High Blood Cholesterol in Adults. Executive Summary of the Third Report of The National Cholesterol Education Program (NCEP) Expert Panel on Detection, Evaluation, and Treatment of High Blood Cholesterol in Adults (Adult Treatment Panel III). JAMA.

[14] Katsouyanni K, Rimm EB, Gnardellis C, Trichopoulos D, Polychronopoulos E, Trichopoulou A (1997). Reproducibility and relative validity of an extensive semi-quantitative food frequency questionnaire using dietary records and biochemical markers among Greek schoolteachers. Int J Epidemiol.

[15] Aebi H (1984). Catalase in vitro. Methods Enzymol.

[16] Paglia DE, Valentine WN (1967). Studies on the quantitative and qualitative characterization of erythrocyte glutathione peroxidase. J Lab Clin Med.

[17] Harrison DG, Gongora MC, Guzic TJ, Widder J (2007). Oxidative stress and hypertension. J Am Soc Hypertens.

[18] Raij L (1998). Nitric oxide in hypertension: relationship with renal injury and left ventricular hypertrophy. Hypertension.

[19] Tsay HJ, Wang P, Wang SL, Ku HH (2000). Age-associated changes of superoxide dismutase and catalase activities in the rat brain. J Biomed Sci.

[20] Isler M, Delibas N, Guclu M, Gultekin F, Sutcu R, Bahceci M (2002). Superoxide dismutase and glutathione peroxidase in erythrocytes of patients with iron deficiency anemia: effects of different treatment modalities. Croat Med J.

[21] Redon J, Oliva MR, Tormos C, Giner V, Chaves J, Iradi A (2003). Antioxidant activities and oxidative stress byproducts in human hypertension. Hypertension.

[22] Chaves FJ, Mansego ML, Blesa S, Gonzales-Albert V, Jimenez J, Tormos MC (2007). Inadequate cytoplasmic antioxidant enzymes response contributes to the oxidative stress in human hypertension. Am J Hypertens.

[23] Simic DV, Mimik-Oka J, Pljesa-Ercegovac M, Savic-Radojevic A, Opacic M, Matic D (2006). Byproducts of oxidative protein damage and antioxidant enzyme activities in plasma of patients with different degrees of essential hypertension. J Hum Hypertens.

[24] Russo C, Olivieri O, Girelli D, Faccini G, Zenari ML, Lombardi S (1998). Anti-oxidant status and lipid peroxidation in patients with essential hypertension. J Hypertens.

[25] Wassmann S, Wassmann K, Nickenig G (2004). Modulation of oxidant and antioxidant enzyme expression and function in vascular cells. Hypertension.

[26] Uchida K, Kawakishi S (1994). Identification of oxidized histidine generated at the active site of Cu, Zn-superoxide dismutase exposed to H_2_O_2_. Selective generation of 2-oxo-histidine at the histidine 118. J Biol Chem.

[27] Lacy F, Kailasam MT, O’Connor DT, Schmid-Schonbein GW, Parmer RJ (2000). Plasma hydrogen peroxide production in human essential hypertension: role of heredity, gender, and ethnicity. Hypertension.

[28] Taddei S, Virdis A, Ghiadoni L, Salvetti G, Bernini G, Magagna A (2001). Age-related reduction of NO availability and oxidative stress in humans. Hypertension.

[29] Goth L (1991). A simple method for determination of serum catalase activity and revision of reference range. Clin Chim Acta.

[30] Rao G, Xia E, Richardson A (1990). Effect of age on the expression of anti-oxidant enzymes in male Fischer F344 rats. Mech Ageing Dev.

[31] Ji LL, Dillon D, Wu E (1991). Myocardial aging: antioxidant enzyme systems and related biochemical properties. Am J Physiol.

